# Volatile Constituents of Some Selected Plant Species Traditionally Used as Tea in the Sharri Mountains (Kosovo)

**DOI:** 10.1155/2022/2594195

**Published:** 2022-05-16

**Authors:** Avni Hajdari, Nita Kelmendi, Genista Mustafa, Behxhet Mustafa, Dashnor Nebija

**Affiliations:** ^1^Department of Biology, Faculty of Mathematical and Natural Science, University of Prishtina “Hasan Prishtina”, Mother Theresa St. 10000, Prishtinë, Kosovo; ^2^Department of Pharmacy, Alma Mater Europaea Campus College “Rezonanca” Glloku te Shelgjet, Prishtina 10000, Kosovo; ^3^Faculty of Medicine, University of Prishtina “Hasan Prishtina”. St. Bulevardi i Dëshmorëve, 10000 Prishtinë, Kosovo

## Abstract

The study evaluates the chemical composition of the volatile constituents of ten plant species traditionally used as herbal tea in the Sharri Mountain regions (Kosovo and North Macedonia). Volatile constituents responsible for the flavour and fragrance of selected species (*Crataegus monogyna*, *Cydonia oblonga, Malus sylvestris*, *Matricaria chamomilla*, *Morus alba, Morus nigra*, *Rosa canina, Sambucus nigra, Tilia cordata,* and *Vaccinium myrtillus*) were separated and then identified using GC-MS, whereas GC-FID is employed for the quantitative analysis. Experimental data revealed different patterns of volatile constituents depending on plant species. Monoterpenes, sesquiterpenes, diterpenes, and norisoprenoids were the most abundant volatile constituents. Principal component analysis (PCA) was deployed for data analysis and resulted in grouping these ten species in four principal clusters. The combination of various volatile constituents present in specific plant species may play an important role in the specific aroma and taste sensation of these species used as recreational teas.

## 1. Introduction

The Sharri Mountain (in Albanian known as Malet e Sharrit; in Macedonian and Serbo-Croatian as Šar Planina) lies in the southern part of Kosovo and northwest part of North Macedonia. The region is rich in terms of biological diversity, and in recognition of its biodiversity richness, a part of Sharri Mountain in Kosovo (1989, extended in 2012) and that in North Macedonia (2021) were declared as a National Park [1–3]. The region possesses valuable cultural heritage, too [1]. The ethnobotanical literature revealed that local communities used local plants as food and as aromatic and refreshing hot beverages (recreational tea) apart from medicinal purposes. Species of the Lamiaceae, Asteraceae, and Rosaceae families were mainly used to prepare herbal tea [4]. In this study, the chemical composition of the volatile constituents of ten local plant species used as recreational tea in Kosovo and North Macedonia parts of the Sharri Region has been evaluated. The studied plants belong to five different families, including Rosaceae (*Crataegus monogyna* Jacq., *Cydonia oblonga* Mill., *Malus sylvestris* (L.) Mill., *and Rosa canina* L.), Adoxaceae (*Sambucus nigra* L.), Ericaceae (*Vaccinium myrtillus* L.), Moraceae *(Morus alba* L. and *Morus nigra* L.), Malvaceae (*Tilia cordata* Mill.), and Asteraceae (*Matricaria chamomilla* L.).

## 2. Materials and Methods

### 2.1. Plant Material

The plant material was collected from July to October 2018 in Sharri Mountains (Kosovo and North Macedonia). Only plant species used as hot aromatic beverages (water infusion) for recreational consumption (excluding those used for specific medical purposes) were selected. They were selected based on the reviewed data from previously published ethnobotanical studies, unpublished ethnobotanical data, and interviews carried out during the fieldwork. Plant material was either collected from wild populations in Sharri Mountains, purchased in the local markets (in 2018), or provided by local residents that were wildcrafted or cultivated for family use. The plant species were identified according to the Flora Europaea [5], while the botanical nomenclature assignments followed The Plant List database [6].

### 2.2. Plant Material Extraction

Plant material was dried, cut into small pieces (>0.3 cm), and then extracted by hydrodistillation (50g of cut plant material in 0.5 litres of deionized water contained in a 1 litre flask) at a distillation rate of 3 ml.min^−1^ using a Clevenger apparatus for 3 hours. Their volatiles were collected in 1 mL *n*-hexane, and the extracts were stored at −18°C in the freezer until further analysis.

### 2.3. GC-MS and GC-FID Analyses

The volatile constituents were separated using a HP-5ms column (30 m × 0.25 mm i.d., film thickness 0.25 mm) in a gas chromatograph (Agilent 7890A). The identification was made using a mass spectrometry detector (Agilent 5975C MSD). The mass spectrometer ionization energy was 70 eV, with a mass range of 40–400 m/z. Helium was used as a carrier gas at an initial flow rate of 0.6 mL/min (50 psi), and 1.0 *μ*L of the sample was injected with a split ratio of 50 : 1. The initial GC oven temperature was 60°C (5 min), increased from 60°C to 280°C at a rate of 5°C/min. GC-FID analyses were performed with the same column and temperature program as the analytical GC/MS.

### 2.4. Identification of Volatile Constituents

Each constituent was identified by comparing the Kovats retention indices with those reported in the literature [7]. The retention arithmetic indices (RIs) were calculated using a linear interpolation of a homologous series of n-alkane (C9–C28) retention times under the same operating conditions. Furthermore, the constituents were identified by comparing the mass spectra of each constituent with those stored in the NIST 08.L and WILEY MS 9th databases. Furthermore, some of the peaks were identified by comparing the retention times and mass spectra with authentic constituents. The relative intensity of each compound has been calculated as the ratio between the area of the specific molecule and the sum of the areas of all identified peaks (peak area normalization method) in the chromatogram [8, 9].

### 2.5. Statistical Analysis

Principal component analysis (PCA) was used for data analyses. Only chemical constituents with concentrations higher than 5% were selected for statistical analysis. The XLStat program (version 2021.2.2) was used for the PCA.

## 3. Results and Discussion

Eighteen plant species were identified for making recreational herbal teas in the Sharri Mountain area. The chemical composition of eight plant species of the Lamiaceae family was reported previously [10], while in this article, the volatile constituents of the species belonging to the families of Adoxaceae, Asteraceae, Ericaceae, Malvaceae, Moraceae, and Rosaceae used for making recreational herbal teas are reported (Table 1). Regarding botanical genera, the genus *Rosa* was represented with four species and the genus *Morus* was represented with two species, while all other genera were represented with only one species.

The volatile composition of the analyzed species is presented in Table 2. Forty-seven volatile compounds were identified in the extracts of the common hawthorn flowers (*Crataegus monogyna* Jacq.). Oxygenated sesquiterpenes (36.35%) were the most prominent constituents, followed by oxygenated monoterpenes (31.22%), sesquiterpenes (18.32%), hydrocarbons (5.74%), fatty acids and derivatives (2.23%), and monoterpenes (1.15%). The principal constituents were spathulenol (22.76%), cis-pinocamphone (19.86%), E-caryophyllene (6.87%), 1.8-cineole (4.52%), caryophyllene oxide (4.17%), and isomenthone (3.17%). In hawthorn leaf and flower samples collected in Turkey, the principal volatile components were aldehydes, benzaldehyde (82.54%), butyraldehyde (38.27%), and (E) 2-hexenal (21.67%), while linoleic (64.23%), oleic (39.36%), and palmitic (8.16%) acids were the principal constituents of seeds [11].

In the extract of the quince leaves (*Cydonia oblonga* Mill.), forty-six compounds were identified. Hydrocarbons (34.44%) and oxygenated sesquiterpenes (23.29%) were the most prominent classes of identified compounds, followed by fatty acids and their derivatives (21.34%) and other compounds (10.61%), whereas sesquiterpenes (5.08%), diterpenes, oxygenated diterpenes (3.55%), and oxygenated monoterpenes were present in smaller amount (0.36%). E-*β*-ionone (7.73%) was the most prominent compound followed by *n*-eicosane (5.94%), 14-oxy-*α*-muurolene (5.38%), *n*-tricosane (4.86%), dodecanoic acid (4.55), 3Z-hexenyl salicylate (4.18%), hexacosane (4.02%), *δ*-cadinol (3.95%), *n*-docosane (3.88%), *n*-hexadecanoic acid (3.67%), *α*-bisabolone oxide (3.62), linoleic acid (3.36%), and 3E-cembrene (3.14%). The principal volatile constituents in quince leaves collected in Turkey during the flowering period were benzaldehyde (12.8%), followed by the fatty acid, hexadecanoic acid (7.2%), oxygenated monoterpene, linalool (5.7%), and E-*β*-ionone (5.1%), while during the fruiting period, the main constituents are sesquiterpenes, germacrene D (8.6%), and benzaldehyde (4.9%) [12]. The percentage of E-*β*-ionone (C13-norisoprenoid) (7.73%) in our samples was higher, whereas the percentage of hexadecanoic acid (3.67%) was lower than in Turkish samples. On the other hand, the most prominent constituents of essential oils obtained from Serbian samples were ethyl 2-methylbutanoate, (E,E)-*α*-farnesene, ethyl-(2E,4Z)-decadienoate, pentadecanol, *β*-acoradienol, ethyl decanoate, ethyl octanoate, (E)-nerolidol, and ethyl dodecanoate [13].

The volatile constituents obtained from European crab apple (*Malus sylvestris* (L). Mill) represent a complex mixture of constituents composed of thirty-seven compounds. The main classes of chemical constituents were sesquiterpenes and oxygenated sesquiterpenes, with 44.87% and 20.11%, respectively (Table 2), followed by other compounds (14.1%), fatty acids and derivatives (8.09%), hydrocarbons (6.88%), and oxygenated monoterpenes (4.62%). The most prominent compounds were E-*β*-farnesene (12.86%), *α*-cadinol (16.6%), E-spiroether (9.7%), *α*-bisabolone oxide (5.9%), *α*-bisabolol oxide A (5.46%), 2E-dodecanal (4.6%), hexacosane (3.51%), and caryophyllene oxide. GC-FID and/or GC-MS analysis of dichloromethane extracts of European wild apple fruit distillates originating from Serbia revealed complex volatile profiles of the main detected components to be alcohols, shikimate metabolites, esters, terpenes, aldehydes and acetyls, fatty acids, and carotenoid-derived compounds [14].

Among the abovementioned species of the family Rosaceae, the volatile constituents were analyzed in the extracts of the dog rose fruits (*Rosa canina* L.) and revealed in the identification of forty-six compounds. Fatty acids and their derivatives (28.56%), hydrocarbons (27.46%), and other compounds, mostly tetrahydrofurans (15.34%), were the principal components in oils, followed by oxygenated monoterpenes (11.59%), sesquiterpenes (6.22%), monoterpenes (4.29%), and oxygenated sesquiterpenes (4.07%). The principal constituents were the fatty alcohol, *n*-tetradecanol (13.51%), followed by a C13 spiroether vitispirane (11.47%), fatty aldehyde, *n-*nonanal (5.12%), oxygenated monoterpene, *α*-terpineol (5.09%), 14-oxy-*α*-muurolene (4.07%), *n-*hexanol (3.86), 1-octadecene (3.69%), (E)-*β*-ocimene (3.55%), heptacosane (3.45%), *n-*octane (3.08%), and *n*-heneicosane (3.01%). In fresh and dried rosehip samples collected in Serbia, diverse compounds were detected, including ketones, *n-*nonanal (5.12%), esters, phenols, sitosterol, and alcohols. Monoterpenes and aromatic acids were present in fresh fruits, while aldehydes, monoterpenes, and aromatic acids were absent in dried rosehip fruits [15].

GC/MS analysis revealed that fifty-six and fifty-one volatile compounds could be identified in the leaves of Morus alba and Morus nigra, respectively (Table 2). The main classes of chemical constituents for *M. alba* are sesquiterpenes (26.52%), followed by hydrocarbons (22.09%), oxygenated sesquiterpenes (18.87%), and diterpenes and oxygenated diterpenes (16.35%). Other compounds (6.23%), oxygenated monoterpenes (4.87%), and fatty acids and derivatives (1.96%) were present in a smaller amount. On the other hand, the main classes of constituents for *M. nigra* are oxygenated diterpenes (32.07%), sesquiterpenes (22.27%), and oxygenated sesquiterpenes (17.2%), followed by hydrocarbons (14.71%), oxygenated monoterpenes (7.07%), and fatty acids and derivatives (2.93%). The principal constituents in *M. alba* oils were trans-phytol (15.71%), germacrene D (6.37%), E-caryophyllene (6.26%), *n*-pentacosane (4.90%), *γ*-eudesmol (4.04%), caryophyllene oxide (3.86%), octacosane (3.76%), squalene (3.44), and *β*-bisabolene (3.2%), while the main constituents in *M. nigra* were trans-phytol (31.78%), germacrene D (6.40%), zierone (4.61%), E-caryophyllene (4.14%), *α*-humulene (3.62%), *n*-pentacosane (3.91%), methyl citronellate (3.31%), octacosane (3.43%), and *γ*-eudesmol (3.39%). In samples collected in Serbia, the principal compound was *trans*-phytol (*M. alba*: 65.4–71.2% and *M. nigra*: 7.9–61.6%). Other classes of prominent compounds were alkanes, carotenoid-derived compounds, and fatty acid-related constituents [16]. Phytol was the most abundant volatile constituent of *M. alba* leaves collected in Hungary [17].

In the essential oil of the chamomile flowers (*Matricaria chamomilla* L.), forty-nine compounds were identified. Oxygenated sesquiterpenes (38.13%) and sesquiterpenes (29.02%) were the principal constituents, followed by other compounds including spiroketals (14.32%), fatty acids and derivatives (5.94%), hydrocarbons (5.64%), diterpenes/oxygenated diterpenes (3.28%), and oxygenated monoterpenes (2.12%). The most abundant characteristic constituents were E-*β*-farnesene (21.17%), *α*-bisabolol oxide A (14.83%), E-spiroether (8.97%), bisabolone oxide (7.53%), epi-*α*-cadinol (4.58%), 2E-dodecenal (4.49%), chamazulene (3.32%), and *trans-*phytol (3.28%). Our findings are in line with previously published data for the most abundant classes of compounds and specific constituents. In samples collected in Serbia and Bosnia and Herzegovina, E-*β*-farnesene, *α*-bisabolol and its oxide, chamazulene, germacrene D, and spiroether were present in high concentrations [18, 19].

The hydrocarbons (49.46%) were the most abundant classes of the volatile compounds in the extracts of elderberry flowers (*Sambucus nigra* L.), followed by fatty acids and their derivatives (21.31%), oxygenated monoterpenes (14.91%), and sesquiterpenes (7.52%) and oxygenated sesquiterpenes. Hydrocarbons, nonadecane (17.72%), *n*-tricosane (10.31%), n-eicosane (5.73%), *n*-heneicosane (4.08%), *n*-pentacosane (5.19%), and *n*-docosane (3.79%) were the most prominent compounds, followed by fatty acids and their derivatives, *n*-hexadecanoic acid (9.94%), linoleic acid (6.28%), and linalool (3.27%). Carvacrol (1.95%), citronellol (1.9%), and methyl citronellate (1.90%) were the most prominent oxygenated monoterpenes, while *trans*-*α*-bergamotene (1.01%) and germacrene D (1.08%) were the principal sesquiterpenes. Our results were in agreement with previously published results of elderberry flower volatile constituents originated from Turkey, dominated by heneicosane (18.8%), tricosane (17.3%), nonadecane (13%), and pentacosane (10.3%) [20]. On the other hand, in samples collected in France, the principal components were hydrocarbons, ethers and oxides, ketones, aldehydes, alcohols, esters, and acids. The major constituents were trans-3,7-dimethyl-l,3,7-octatrien-3-ol, palmitic acid (11.3%), linalool (3.7%), cis-hexenol, and cis- and trans-rose oxides [21]. In comparison with our results, linalool percentages detected in our sample were similar to those samples originated from France (3.7%).

Twenty-four volatile compounds were identified in European blueberry (*Vaccinium myrtillus* L.) fruit extracts. Oxygenated monoterpenes (59.65%) were the most prominent constituents, followed by fatty acids and their derivatives (17.38%), sesquiterpenes (11.79%), and oxygenated sesquiterpenes (4.28%). Oxygenated diterpenes and hydrocarbons were present in smaller concentrations. Oxygenated monoterpenes, neral (31.24%) and geranial (22.31%), were the most prominent compounds, followed by sesquiterpene E-caryophyllene (10.73%) and its oxygenated derivative, and caryophyllene oxide (3.85%). Peak areas of hexadecanoic (palmitic) acid, 3Z-hexenyl salicylate, dodecanoic (lauric) acid, and linoleic acid were 9.06%, 2.98%, 2.40%, and 2.12%, respectively. To the best of our knowledge, only a few papers reported data on the chemical composition of the volatile constituents obtained from the fruits of the European blueberry. In the study reported by Jens et al. [22], 99 volatile compounds were identified in blueberry based on MS data, including alkanes, acids, alcohols, aldehydes, esters, ketones, and mono- and sesquiterpenes. On the other hand, HS-SPME analyses revealed complex volatile profiles, including terpenes such as p-cymene, 1,8-cineole, linalool, and aromatic compounds, which contribute to the characteristic blueberry aroma.

In the leaf extracts of small-leaved lime (*Tilia cordata* Mill.), seventy compounds were identified, predominantly belonging to chemical classes of hydrocarbons (44.36%), fatty acids and their derivatives (22.99%), oxygenated monoterpenes (7.5%), oxygenated sesquiterpenes (5.41%), and sesquiterpenes (3.18%). The most prominent class of compounds was hydrocarbons: *n-*pentacosane (11.66%), squalene (4.61%), *n*-nonacosane (2.78%), *n*-heneicosane (11.49%), and *n*-tricosane (4.72%), followed by fatty acids and their derivatives, including *n-*nonanal (7.15%), hexadecanol (2.93%), and dodecanoic acid (2.24%). The concentration of allylbenzene derivative, eugenol and terpenoid ketone, and Z-jasmone, was 6.78% and 3.08%, respectively. In accordance with our data, the study of essential oil from the flowers, bracts, and leaves of *Tilia* sp. originated from Turkey showed high percentages of hydrocarbons and fatty acids and their derivatives (47.5–66.5% in comparison with 49.46% in our sample). Likewise, the high content of aliphatic acids was found in samples from Turkey (28.3–37.1%) [23].

### 3.1. Statistical Analysis

**Figure 1:**
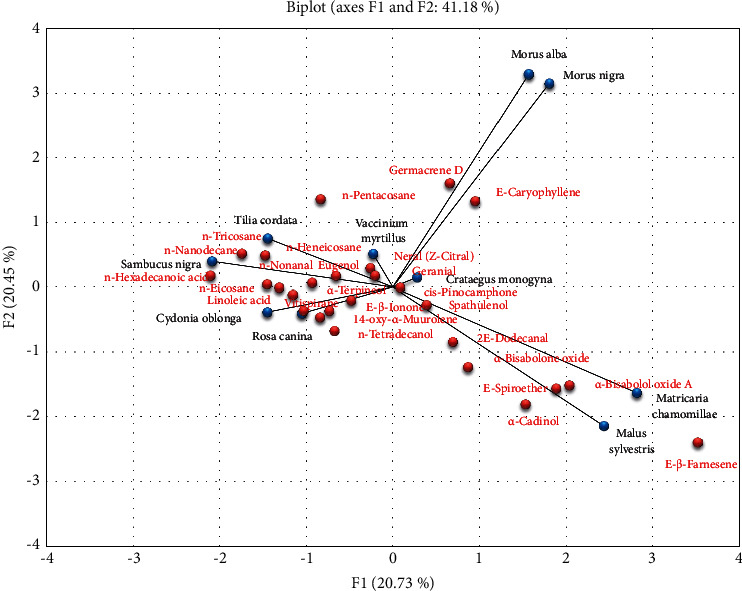
Diagram generated from the principal component analysis of volatile compounds found in ten plant species traditionally used as teas.

Principal component analysis (PCA) has been used to group the analyzed plant species based on their chemical composition (Figure 1). PCA demonstrated that the analyzed plant species had been grouped into four principal groups: *Tilia cordata*, *Sambucus nigra,* and *Vaccinium myrtillus* clustered in the first group, *Morus alba*, *Morus nigra,* and *Crataegus monogyna* clustered in the second group, and *Matricaria chamomilla* and *Malus sylvestris* clustered in the third group, whereas the fourth group included *Cydonia oblonga* and *Rosa canina*, with greater similarities concerning their chemical composition (Figure 1).

### 3.2. Frequency of the Used Species as Tea

Some of the plant species analyzed in this study still have importance in use as herbal in the culture of the Sharri Region. Thus, *Matricaria chamomilla, Tilia cordata,* and *Rosa canina* are still frequently used in this region, and Vaccinium myrtillus *is* occasionally used to prepare tea. The species *Malus sylvestris* and *Sambucus nigra* are rarely used nowadays, while *Morus alba*, *Morus nigra*, *Crataegus monogyna,* and *Cydonia oblonga* are not used anymore for this purpose.

## 4. Conclusions

Eighteen plant species were identified to be used as recreational herbal teas in the Sharri Mountain area. The chemical composition of eight plant species (Lamiaceae family) was reported previously, while in this article, the volatile constituents of the species belonging to the families of Adoxaceae, Asteraceae, Ericaceae, Malvaceae, Moraceae, and Rosaceae are reported. Experimental data revealed different patterns of volatile constituents depending on plant species. The variety of volatile constituents present in plant species suggests that their combination may play an important role in the specific aroma and taste sensation of recreational teas, and these factors are important for consumers' preferences. On the other hand, besides phytochemical characteristics, evaluating the pharmacological and nutritional properties of plants used for the tea preparation is needed to warrant their safe and appropriate use, especially for those teas consumed regularly.

## Figures and Tables

**Table 1:** List of the selected plant species used for tea preparation.

Plant family	Plant species	Plant organs used	Plant origin	Frequency of use
Asteraceae	*Matricaria chamomilla* L.	Flowers	Wild	Still being used
Malvaceae	*Tilia cordata* Mill.	Leaves	Wild	Still being used
Rosaceae	*Rosa canina L.*	Fruits	Wild	Still being used
Ericaceae	*Vaccinium myrtillus* L.	Fruits	Wild	Occasionally used
Rosaceae	*Malus sylvestris Mill.*	Fruits	Wild	Rarely used
Adoxaceae	*Sambucus nigra L.*	Flowers	Wild	Rarely used
Moraceae	*Morus alba L.*	Leaves	Cultivated	No longer used
Moraceae	*Morus nigra* L.	Leaves	Cultivated	No longer used
Rosaceae	*Crataegus monogyna* Jacq.	Flowers	Wild	No longer used
Rosaceae	*Cydonia oblonga* Mill.	Leaves	Cultivated	No longer used

**Table 2:** Volatile compounds of essential oils obtained from the extracts of ten plants used as teas.

Compound name and class^a^	RI^b^	ID^c^	Rosaceae				Moraceae		Asteraceae	Adoxaceae	Ericaceae	Malvaceae
			*C. monogyna*	*C. oblonga*	*M. sylvestris*	*R. canina*	*M. alba*	*M. nigra*	*M. chamomilla*	*S. nigra*	*V. myrtillus*	*T. cordata*
*n*-Octane	800	1,2	—	—	—	3.08	—	—	—	—	—	—
(2E)-hexenal	846	1,2	—	—	—	—	—	—	—	—	—	1.27
(2E)-hexenol	854	1,2	—	—	—	0.46	—	—	—	—	—	0.12
*n-*Hexanol	870	1,2	—	—	—	3.86	—	—	—	—	—	—
n-Nonane	900	1,2	—	—	—	—	—	—	—	—	—	0.18
Heptanal	902	1,2	—	—	—	0.33	—	—	—	0.14	—	1.17
*α*-Pinene	932	1,2,3	—	—	—	0.35	—	—	—	—	—	—
Camphene	946	1,2,3	—	—	—	0.39	—	—	—	—	—	—
Benzaldehyde	952	1,2	—	—	—	—	—	—	—	—	—	0.22
*β*-Pinene	974	1,2,3	0.68	—	—	—	—	—	—	—	—	0.47
3-Octanone	979	1,2	—	—	—	—	—	—	—	—	—	0.25
1-Decene	986	1,2	—	—	—	2.57	—	—	0.14	—	—	0.46
*p*-Cymene	1020	1,2	—	—	—	—	—	—	0.22	0.06	—	0.61
Limonene	1024	1,2,3	—	—	—	—	0.42	0.42	—	—	—	—
1.8-Cineole	1026	1,2,3	4.52	—	—	—	—	—	—	—	—	—
Benzene acetaldehyde	1036	1,2	—	—	—	1.73	—	—	—	—	0.15	1.26
(E)-*β*-ocimene	1044	1,2	0.47	—	—	3.55	—	—	0.17	—	—	—
*n*-Octanol	1063	1,2	—	—	—	0.45	—	—	1.06	—	—	0.70
Linalool	1095	1,2	—	—	—	1.32	—	—	—	3.27	1.12	—
trans-Sabinene hydrate	1098	1,2	—	—	—	—	0.89	0.85	—	1.00	—	—
*n*-Nonanal	1099	1,2	—	—	—	**5.12**	—	—	—	—	—	**7.15**
cis-Rose oxide	1106	1,2	—	—	—	—	—	—	—	0.66	—	—
trans-Rose oxide	1122	1,2	—	—	—	—	—	—	—	0.75	—	—
trans-Pinocarveol	1135	1,2	1.29	—	—	—	—	—	—	—	—	—
Camphor	1141	1,2,3	—	—	—	—	—	—	—	0.17	0.17	—
Citronellal	1148	1,2	—	—	—	—	—	—	—	—	1.22	—
Nerol oxide	1154	1,2	—	—	—	—	—	—	—	0.29	—	—
Isomenthone	1158	1,2	3.17	—	—	1.10	0.62	0.49	—	—	—	0.36
Borneol	1165	1,2,3	0.67	—	—	—	—	—	—	0.05	1.75	0.70
cis-Pinocamphone	1172	1,2,3	**19.86**	—	—	—	—	—	—	—	0.22	0.20
*α*-Terpineol	1186	1,2,3	0.32	0.36	—	**5.09**	—	—	—	0.15	—	0.73
2-Decanone	1190	1,2	1.72	—	—	—	—	—	—	—	—	0.70
*n*-Decanal	1201	1,2	—	—	—	0.41	0.35	1.26	—	—	—	1.82
Citronellol	1223	1,2,3	—	—	—	1.37	—	—	—	1.90	—	0.34
Pulegone	1233	1,2,3	—	—	—	0.39	—	—	0.31	0.24	—	0.78
trans-Chrysanthenyl acetate	1235	1,2	—	—	—	—	—	—	—	0.71	—	—
Neral (Z-citral)	1235	1,2,3	—	—	—	—	—	—	—	—	**31.24**	—
Carvone	1239	1,2	0.35	—	—	—	—	—	—	—	—	—
Geraniol	1249	1,2,3	—	—	—	—	—	—	0.74	—	1.11	—
Methyl citronellate	1257	1,2	0.47	—	—	—	2.04	3.31	—	1.90	—	0.87
Chrysanthenyl acetate	1261	1,2	—	—	0.87	0.38	—	—	0.17	—	—	—
Geranial	1264	1,2	0.28	—	—	—	—	—	—	—	**22.31**	—
Nonanoic acid	1267	1,2	—	0.26	—	0.37	—	—	—	—	—	0.57
Vitispirane	1279	1,2	—	—	—	**11.47**	—	—	—	—	—	—
Thymol	1289	1,2	—	—	—	—	—	—	—	0.42	—	—
(2E,4E)-Decadienal	1293	1,2	—	—	—	0.35	—	—	—	0.94	—	—
Carvacrol ethyl ether	1297	1,2	—	—	0.57	—	0.60	0.67	—	—	—	—
Carvacrol	1299	1,2	—	—	—	1.25	—	—	0.16	1.95	—	—
Undecanal	1306	1,2	—	—	—	0.49	—	—	—	—	—	1.47
2-Adamantanone	1311	1,2	—	—	—	—	—	—	—	—	—	0.23
cis-Dihydro-*α*-terpinyl acetate	1316	1,2	—	—	—	0.69	—	—	—	0.40	—	—
Methyl geranate	1322	1,2	—	—	—	—	0.47	0.91	—	0.42	0.68	1.89
Z-Hasmigone	1327	1,2	—	—	—	—	—	—	—	—	—	0.28
*α*-Terpinyl acetate	1346	1,2	—	—	—	—	—	—	—	0.27	—	—
Eugenol	1356	1,2,3	—	—	—	—	—	—	—	0.49	—	**6.78**
Decanoic acid	1364	1,2	—	0.67	0.70	0.84	—	—	—	—	0.82	0.28
*α*-E-ionol	1376	1,2	—	—	—	1.88	—	—	—	—	—	0.55
Geranyl acetate	1379	1,2,3	—	—	1.01	—	0.25	0.84	0.23	0.19	—	—
E-*β*-damascenone	1384	1,2	—	1.49	—	—	—	—	0.22	—	—	—
*β*-Bourbonene	1388	1,2	2.41	—	1.02	—	0.40	1.00	1.19	—	—	—
*β*-Elemene	1390	1,2	0.35	—	—	—	—	—	—	—	—	0.21
Z-jasmone	1392	1,2	—	0.26	—	—	0.52	0.39	—	—	—	3.08
Sibirene	1400	1,2	—	—	—	1.87	1.98	0.48	—	0.60	—	—
Methyl eugenol	1403	1,2	—	—	—	—	—	—	0.82	0.54	—	0.70
*α*-Cedrene	1409	1,2,3	—	—	1.40	—	0.49	—	—	0.98	—	—
E-caryophyllene	1417	1,2,3	**6.87**	0.31	2.04	—	**6.26**	4.14	1.22	0.28	**10.73**	0.22
*β*-Ylangene	1420	1,2	—	—	—	0.49	—	—	—	—	—	0.60
E-*α*-ionone	1428	1,2	—	—	—	—	0.31	1.07	—	0.27	—	0.37
trans-*α*-Bergamotene	1432	1,2	—	—	—	—	—	—	—	1.01	—	—
Cedrane	1441	1,2	0.28	—	—	0.49	—	—	—	—	—	0.30
*α*-Humulene	1452	1,2,3	1.67	0.82	—	—	2.79	3.62	—	—	0.38	0.12
E-*β*-farnesene	1454	1,2,3	—	—	**12.86**	0.66	0.32	1.52	**21.17**	0.39	—	—
Allo-aromadendrene	1458	1,2	1.01	—	—	—	—	—	—	0.28	—	—
2E-dodecenal	1464	1,2	—	1.27	4.60	—	—	—	4.49	—	—	0.36
Germacrene D	1480	1,2,3	2.87	0.98	0.88	—	**6.37**	**6.40**	—	1.08	—	0.84
E-*β*-ionone	1487	1,2	1.42	**7.73**	1.67	0.26	0.24	—	—	0.11	—	0.17
*β*-Vetispirene	1489	1,2	—	—	—	—	0.31	0.82	0.82	—	—	0.22
*n*-Pentadecane	1500	1,2,5	—	1.28	—	—	—	—	0.43	—	—	—
Bicyclogermacrene	1500	1,2	0.46	—	1.77	—	2.48	0.94	1.08	0.37	—	—
Piperonyl acetate	1503	1,2	—	—	1.38	—	2.75	—	0.17	—	—	0.23
*β*-Bisabolene	1505	1,2	—	0.39	0.14	—	3.20	1.10	0.25	0.42	—	—
Germacrene A	1509	1,2	—	—	—	2.71	—	—	—	—	—	—
*δ*-Amorphene	1512	1,2	—	—	—	—	1.14	1.00	—	0.64	—	0.44
*γ*-Cadinene	1513	1,2	—	0.50	—	—	—	—	—	0.51	—	—
*δ*-Cadinene	1522	1,2	2.40	2.08	—	—	1.09	2.07	0.28	0.96	—	0.45
10-epi-Cubebol	1533	1,2	—	—	1.05	—	—	—	1.80	—	—	0.28
Hedycaryol	1546	1,2	—	—	—	—	1.04	0.47	1.58	—	—	0.23
E-Isocroweacin	1553	1,2	—	—	—	—	2.10	1.22	—	—	—	—
Germacrene B	1559	1,2	—	—	—	—	—	—	0.45	—	—	—
Geranyl butyrate	1562	1,2	0.29	—	2.17	—	—	—	0.51	0.34	—	1.63
Dodecanoic acid	1566	1,2	—	4.55	—	0.78	—	—	—	—	2.40	2.24
Zierone	1575	1,2	—	—	0.44	—	1.34	4.61	1.62	—	—	—
Spathulenol	1577	1,2	**22.76**	0.43	2.07	—	—	—	1.10	0.26	—	—
Caryophyllene oxide	1583	1,2	4.17	0.54	2.73	—	3.86	1.25	0.73	0.29	3.85	—
Globulol	1590	1,2	2.56	—	—	—	0.79	—	—	—	—	—
Humulene epoxide II	1608	1,2	1.89	1.11	—	—	1.54	—	—	—	0.43	—
Tetradecanal	1612	1,2	—	—	—	—	—	—	—	0.58	—	0.67
Isolongifolan-7-*α*-ol	1619	1,2	—	—	1.42	—	1.24	—	—	—	—	—
*γ*-Eudesmol	1630	1,2	0.59	—	—	—	4.04	3.39	—	0.31	—	—
Epi-*α*-cadinol	1638	1,2	—	—	1.41	—	—	—	4.58	—	—	—
*α*-Muurolol	1644	1,2	—	0.76	0.72	—	1.37	1.57	—	—	—	0.20
*β*-Eudesmol	1649	1,2	—	—	—	—	0.34	0.63	1.50	0.27	—	—
*α*-Eudesmol	1652	1,2	0.55	—	1.11	—	1.32	1.06	1.38	—	—	—
*α*-Cadinol	1654	1,2,3	0.37	3.95	**16.60**	—	0.55	0.46	2.69	0.33	—	0.17
Intermedeol	1665	1,2	1.93	2.06	—	—	0.37	0.42	—	—	—	—
6Z-coniferyl alcohol	1668	1,2	0.38	—	—	—	—	—	—	—	—	0.46
3Z-hexenyl salicylate	1669	1,2	1.36	4.18	—	—	0.36	0.36	—	—	2.98	—
*n*-Tetradecanol	1672	1,2	—	0.79	2.55	**13.51**	—	—	—	—	—	—
Guaia-3,10(14)-diene-11-ol	1677	1,2	0.20	—	—	—	0.41	1.18	—	—	—	—
Khusinol	1680	1,2	—	—	1.37	—	—	—	—	—	—	—
Elemol acetate	1680	1,2	—	—	1.75	—	—	—	—	—	—	—
*α*-Bisabolone oxide	1685	1,2	—	3.62	**5.90**	—	—	—	**7.53**	0.23	—	—
Eudesma-4(15),7-dien-1*β*-ol	1688	1,2	0.94	—	—	—	—	—	0.94	—	—	—
n-Heptadecane	1700	1,2	—	1.15	0.78	—	0.36	0.63	2.05	0.67	—	1.37
E-nerolidyl acetate	1717	1,2	—	0.89	—	—	—	0.58	0.58	—	—	—
2Z,6E-farnesol	1723	1,2	—	—	2.00	—	—	—	0.45	—	—	—
Chamazulene	1731	1,2	—	1.13	1.35	—	—	—	3.32	—	—	—
2E,6E-farnesal	1741	1,2	—	0.92	—	—	—	—	—	—	—	0.12
*α*-Bisabolol oxide A	1748	1,2	—	—	**5.46**	—	—	—	**14.83**	—	—	—
14-Oxy-*α*-muurolene	1766	1,2	—	**5.38**	0.84	4.07	—	—	—	—	0.68	1.97
2-*α*-hydroxy-amorpha-4,7(11)-diene	1776	1,2	0.39	1.95	—	—	—	—	—	—	—	—
*γ*-Eudesmol acetate	1783	1,2	—	0.84	—	—	—	—	0.20	—	—	0.58
1-Octadecene	1790	1,2	—	2.70	—	3.69	—	—	0.41	—	—	—
Cyclopentadecanolide	1833	1,2	—	0.50	—	—	1.01	0.30	0.14	—	—	0.63
Phenyl ethyl octanoate	1847	1,2	—	—	—	—	—	—	—	—	—	0.66
Unidentified	1850	1,2	—	—	—	0.99	—	—	—	0.99	—	—
Z,Z-farnesyl acetone	1861	1,2	—	—	—	—	0.45	0.66	—	0.70	—	—
n-Hexadecanol	1875	1,2	—	—	—	—	—	—	—	0.95	—	2.93
E-spiroether	1890	1,2	—	—	**9.70**	—	—	—	**8.97**	—	—	—
*n*-Nonadecane	1900	1,2	—	2.51	—	—	—	—	—	**17.72**	—	1.51
5E,9E-farnesyl acetone	1912	1,2	—	0.84	—	—	0.21	0.36	—	—	—	1.86
Cyclohexadecanolide	1934	1,2	—	—	—	—	—	—	—	1.15	—	—
3E-cembrene A	1947	1,2	—	3.14	—	—	—	—	—	—	—	—
n-Hexadecanoic acid	1959	1,2	—	3.67	—	—	—	—	0.25	9.94	9.06	—
*n*-Eicosane	2000	1,2	—	5.94	—	1.84	1.23	0.14	—	5.73	—	—
6Z,10E-pseudo phytol	2030	1,2	—	—	—	—	0.64	0.29	—	—	—	—
*n*-Heneicosane	2100	1,2	0.28	—	—	3.01	0.42	0.38	—	4.08	0.97	11.49
trans-Phytol	2122	1,2	0.27	0.41	—	—	15.71	31.78	3.28	—	1.41	—
Linoleic acid	2133	1,2	0.54	3.36	—	1.60	—	—	—	6.28	2.12	—
Oleic acid	2142	1,2	0.33	2.09	0.24	—	0.24	0.35	—	1.91	—	—
*n*-Docosane	2200	1,2	0.66	3.88	0.16	1.70	0.56	0.30	0.29	3.79	—	—
*n*-Tricosane	2300	1,2	0.84	4.86	0.20	0.30	1.85	1.06	—	10.31	—	4.72
*n*-Tetracosane	2400	1,2	0.29	1.50	0.18	0.92	2.23	1.72	0.41	1.39	—	1.08
*n*-Pentacosane	2500	1,2	0.22	2.44	2.05	1.20	4.90	3.91	1.43	**5.19**	1.88	**11.66**
Hexacosane	2600	1,2	0.84	4.02	3.51	2.86	0.92	0.43	—	—	—	1.29
Heptacosane	2700	1,2	1.27	2.42	—	3.45	2.42	1.81	0.48	—	0.81	2.03
Squalene	2785	1,2	—	—	—	—	3.44	0.90	—	—	—	4.61
Octacosane	2800	1,2	1.34	1.74	—	2.84	3.76	3.43	—	—	—	1.18
Nonacosane	2900	1,2	—	—	—	—	—	—	—	—	—	2.78
Total			98.8	98.67	98.67	98.53	97.31	98.95	98.84	98.22	98.49	99.07
Monoterpenes			1.15	—	—	4.29	0.42	0.42	0.39	0.23	0.17	1.08
Oxygenated monoterpenes			31.22	0.36	4.62	11.59	4.87	7.07	2.12	14.91	59.65	7.5
Sesquiterpenes			18.32	5.08	20.11	6.22	26.52	22.27	29.02	7.52	11.79	3.18
Oxygenated sesquiterpenes			36.35	23.29	44.87	4.07	18.87	17.2	38.13	2.39	4.28	5.41
Diterpenes, oxygenated diterpenes			0.27	3.55		—	16.35	32.07	3.28	—	1.41	—
Fatty acids and derivatives			2.23	21.34	8.09	28.56	1.96	2.93	5.94	21.31	17.38	22.99
Hydrocarbons			5.74	34.44	6.88	27.46	22.09	14.71	5.64	49.46	3.66	44.36
Other compounds			3.52	10.61	14.1	15.34	6.23	2.28	14.32	1.41	0.15	14.55
Unidentified			—	—	—	0.99	—	—	—	0.99	—	—

^a^Compounds listed in the order of elution from an HP-5ms column. ^b^RI = Retention arithmetic index calculated against a mixture of C9–C28n alkanes. ^c^ID = Peak identification mode: 1. identified by comparison of mass spectra; 2. identified by retention index matching; and 3. identified by comparing the constituent retention times with authentic constituents. The percentage for each population represents the average values of three samples. Compounds marked in boldface (with concentrations higher than 5%) were chosen for PCA statistical analyses. — = trace <0.1%.

## Data Availability

The GC-MS data used to support the findings of this study are available from the corresponding author upon request.

## Abbreviations

GC-FID: Gas chromatography-flame ionization detection
GC-MS: Gas chromatography-mass spectrometry
PCA: Principal component analyses.
